# Dexmedetomidine Alleviates Hypoxia-Induced Synaptic Loss and Cognitive Impairment via Inhibition of Microglial NOX2 Activation in the Hippocampus of Neonatal Rats

**DOI:** 10.1155/2021/6643171

**Published:** 2021-02-12

**Authors:** Xiaohui Chen, Dongtai Chen, Qiang Li, Shuyan Wu, Jiahao Pan, Yanling Liao, Xiaochun Zheng, Weian Zeng

**Affiliations:** ^1^Department of Anesthesiology, Shengli Clinical Medical College of Fujian Medical University, Fujian Provincial Hospital, Fuzhou, China; ^2^Department of Anesthesiology, Sun Yat-Sen University Cancer Center, State Key Laboratory of Oncology in South China, Collaborative Innovation Center for Cancer Medicine, Guangzhou, China

## Abstract

**Background:**

Perinatal hypoxia is a universal cause of death and neurological deficits in neonates worldwide. Activation of microglial NADPH oxidase 2 (NOX2) leads to oxidative stress and neuroinflammation, which may contribute to hypoxic damage in the developing brain. Dexmedetomidine has been reported to exert potent neuroprotection in several neurological diseases, but the mechanism remains unclear. We investigated whether dexmedetomidine acts through microglial NOX2 to reduce neonatal hypoxic brain damage.

**Methods:**

The potential role of microglial NOX2 in dexmedetomidine-mediated alleviation of hypoxic damage was evaluated in cultured BV2 microglia and neonatal rats subjected to hypoxia. *In vivo*, neonatal rats received dexmedetomidine (25 *μ*g/kg, i.p.) 30 min before or immediately after hypoxia (5% O_2_, 2 h). Apocynin-mediated NOX inhibition and lentivirus-mediated NOX2 overexpression were applied to further assess the involvement of microglial NOX2 activation.

**Results:**

Pre- or posttreatment with dexmedetomidine alleviated hypoxia-induced cognitive impairment, restored damaged synapses, and increased postsynaptic density-95 and synaptophysin protein expression following neonatal hypoxia. Importantly, dexmedetomidine treatment suppressed hypoxia-induced microglial NOX2 activation and subsequent oxidative stress and the neuroinflammatory response, as reflected by reduced 4-hydroxynonenal and ROS accumulation, and decreased nuclear NF-*κ*B p65 and proinflammatory cytokine levels in cultured BV2 microglia and the developing hippocampus. In addition, treating primary hippocampal neurons with conditioned medium (CM) from hypoxia-activated BV2 microglia resulted in neuronal damage, which was alleviated by CM from dexmedetomidine-treated microglia. Moreover, the neuroprotective effect of dexmedetomidine was reversed in NOX2-overexpressing BV2 microglia and diminished in apocynin-pretreated neonatal rats.

**Conclusion:**

Dexmedetomidine targets microglial NOX2 to reduce oxidative stress and neuroinflammation and subsequently protects against hippocampal synaptic loss following neonatal hypoxia.

## 1. Introduction

Hypoxic damage to the developing brain remains a universal cause of death and neurodevelopmental disorders in human neonates, affecting approximately 1-3/1000 live births in developed countries [1], and the incidence in certain developing countries is up to 10.7% [2]. Patients surviving neonatal hypoxia commonly experience long-term neurological deficits, including cerebral palsy, seizures, and cognitive impairment [3, 4]. To date, there are still very few preventative or protective therapies available for neonates subjected to acute hypoxic insults [5]. Thus, in-depth exploration of the underlying mechanisms of hypoxic brain damage and identification of new effective therapies are important and urgent issues.

Accumulating evidence has indicated that microglial activation and subsequent neuroinflammation play an important role in the pathogenesis of brain damage following neonatal hypoxia [5–7]. In response to hypoxia, microglia are rapidly activated, which can then result in the production of neurotoxic mediators, including glutamate, interleukin-6 (IL-6), tumor necrosis factor-*α* (TNF-*α*), reactive oxygen species (ROS), and inducible NO synthase (iNOS) which can collectively induce neuronal damage and synaptic loss in the developing brain, especially in the hippocampus [8–10]. Synaptic proteins are key regulators of dendritic spine morphogenesis and plasticity, which are essential for memory and cognitive function [11]. Numerous clinical and experimental studies have shown that neuroinflammation can impair synaptic transmission and plasticity in various neurodegenerative diseases [12–14]. This damage could contribute to long-term cognitive deficits in neonates. Given these findings, suppression of microglia-related neuroinflammation has been regarded as an effective strategy to treat hypoxic brain damage.

NADPH oxidase 2 (NOX2, also called gp91^phox^), a multisubunit enzyme, is a major source of ROS that contributes to the pathology of both acute brain damage and chronic neurodegenerative diseases [15, 16]. It has been reported that NOX2 is localized in many cell types, including neurons and neutrophils, and is mainly localized in microglia, where it is a key regulator of neurotoxic microglial activation [17, 18]. When microglial NOX2 is activated, several ROS, such as superoxide anion (O_2_^−^), hydrogen peroxide (H_2_O_2_), and hydroxyl radicals (OH^−^), are produced that can directly cause oxidative damage to neighboring neurons through extracellular pathways, and intracellular ROS in microglia can enhance the generation of proinflammatory and neurotoxic factors, leading to excessive neuroinflammation [19, 20]. Many studies, including our own, have shown that pharmacological inhibition or genetic deletion of NOX2 can considerably attenuate inflammation, reduce oxidative stress damage, and improve neurological function in the contexts of brain damage and neurodegenerative diseases [18, 21, 22]. Accordingly, by focusing on microglial NOX2 activation following neonatal hypoxia, a promising therapeutic target for improving synapse impairment may be achieved.

Dexmedetomidine is a highly selective *α*-2 adrenergic receptor agonist with sedative, analgesic, and anxiolytic properties that is widely used in the perioperative period and intensive care units [23]. An increasing number of studies have revealed that dexmedetomidine exhibits neuroprotective activities in numerous neurological disorders, including Alzheimer's disease, traumatic brain injury (TBI), and cerebral ischemia [24–26]. Notably, dexmedetomidine has also been found to exert potent anti-inflammatory effects that improve neurological outcomes in a neonatal model of brain hypoxic-ischemia [27]. In our previous work, we found that dexmedetomidine pretreatment protected differentiated *PC12* cells against chemical hypoxia-induced apoptosis, which was associated with the inhibition of NOX2-derived oxidative stress [28]. However, very little is known about the potential of dexmedetomidine to improve neonatal hypoxia-induced cognitive impairment and whether such protection might be associated with inhibition of microglial NOX2 activation and mediation of synaptic plasticity.

Therefore, in this study, we investigated the effects of dexmedetomidine (administered before or after the hypoxic phase) on neonatal hypoxic brain damage and further explored the potential molecular mechanisms involved in this process.

## 2. Materials and Methods

### 2.1. Animals

This study was approved by the Institutional Animal Care and Use Committee of Sun Yat-Sen University Cancer Center (Guangdong, China). The animal experiments were performed in accordance with the Guide for the Care and Use of Laboratory Animals (National Research Council Committee, 2011). Nursing Sprague–Dawley (SD) rats and their offspring were obtained from the Experimental Animal Center of Sun Yat-Sen University. The rats were kept under a 12 h light–dark cycle at 20–22°C with free access to food and water.

### 2.2. Neonatal Hypoxia and Treatment In Vivo

On postnatal day 3 (P3), rat pups (both sexes) were kept in a hypoxia chamber (Model: MCO 18 M; Sanyo Biomedical Electrical Co., Ltd., Tokyo, Japan) filled with humidified 5% O_2_ and 95% N_2_ at 37°C for 2 h [29]. The pups were then allowed to recover under normoxic conditions and returned to their home cages for 1 day or 28 days before euthanasia. Meanwhile, some littermates were kept outside of the chamber at 37°C and used as matched controls.

The rat pups were randomly divided into four groups subjected to different treatments: (1) normoxia + vehicle (saline) (the Control group), (2) hypoxia + vehicle (saline) (the Hypoxia group), (3) hypoxia + dexmedetomidine pretreatment (the Hypo+Pre-Dex group), and (4) hypoxia + dexmedetomidine posttreatment (the Hypo+Post-Dex group). Dexmedetomidine (Dex, 25 *μ*g/kg, Sigma-Aldrich, St. Louis, MO, USA) was intraperitoneally administered 30 min before hypoxia (pre-Dex) or immediately after hypoxic insult (post-Dex). The administration dose and route of dexmedetomidine were chosen according to previous neuroprotection studies [27, 30]. The control group received the same volume of saline as the vehicle. The levels of synapse-associated proteins (synaptophysin and PSD-95), inflammatory cytokines, oxidative stress, and microglial NOX2 in the hippocampus were measured 1 day after neonatal hypoxia. Cognitive function, synaptic ultrastructure and synapse-associated protein levels were measured 28 days after neonatal hypoxia.

Moreover, to specifically confirm the role of NOX2 in the protective effect of dexmedetomidine on hypoxic brain damage, the NOX-specific inhibitor apocynin was used in our further experiments. Apocynin was first dissolved in DMSO and then diluted in saline (final DMSO concentration, <1%). Apocynin (50 mg/kg-) was intraperitoneally injected, followed by dexmedetomidine 30 min later. The administration dosage and route of apocynin were chosen according to a previous study [22].

### 2.3. BV2 Microglial Cell Culture and Treatment In Vitro

The BV2 microglial cell lines were purchased from the Cell Resource Center of the Shanghai Institutes for Biological Sciences (Shanghai, China). BV2 microglia were maintained in DMEM medium (Invitrogen, Carlsbad, CA, USA) containing 10% fetal bovine serum (FBS, Gibco Industries Inc., Grand Island, NY, USA), 100 *μ*g/ml streptomycin, 100 U/ml penicillin, and 2 mM L-glutamine (Invitrogen, Carlsbad, CA, USA) at 37°C in a humidified 5% CO_2_ incubator. BV2 microglia, which are immortalized microglial cells, have been widely used as substitutes for primary microglial cells in many neuroinflammatory experiments, including complex cell-cell interaction studies [31].

For *hypoxia* treatment, BV2 microglia were placed in a hypoxia chamber (model: MCO 18 M) filled with humidified 1% O_2_, 5% CO_2_, and 94% N_2_ at 37°C for 12 h and then cultured under a normoxic atmosphere of 5% CO_2_ and 95% air for 24 h. Meanwhile, BV2 microglia cultured under normoxic atmosphere were used as controls [29]. The cells were also randomly divided into four groups subjected to different treatments: (1) normoxia + vehicle (saline) (the Control group), (2) hypoxia + vehicle (saline) (the Hypoxia group), (3) hypoxia + pre-Dex (the Hypo+Pre-Dex group), and (4) hypoxia + post-Dex (the Hypo+Post-Dex group). BV2 microglia were treated with 1 *μ*M dexmedetomidine 2 h before hypoxia (pre-Dex) or immediately after hypoxic insult (post-Dex). The concentration and timing of dexmedetomidine were chosen based on our previous study [28]. The Control group was treated with sterile saline as the vehicle. The levels of inflammatory cytokines, oxidative stress, and NOX2 activity and expression in BV2 microglia, were measured 24 h following hypoxia.

### 2.4. Overexpression of NOX2 in BV2 Microglia

A lentivirus that overexpressed the NOX2 gene was constructed (Jikai Gene Technology Co., Ltd., Shanghai, China) to further study the role of microglial NOX2 activation in hypoxic neuronal damage. The lentivirus overexpressing NOX2 was termed NOX2, and the lentivirus containing the control vector was termed Vector. BV2 microglia that were not transfected with lentiviruses served as blank controls. BV2 microglial cell lines stably overexpressing NOX2 were established according to the manufacturer's instructions (Jikai Gene, Shanghai, China). The infection efficiency of the vector and NOX2 lentiviruses was confirmed by Western blot analysis.

### 2.5. Conditioned Medium (CM) of BV2 Microglia

To evaluate bystander damage of immature hippocampal neurons induced by microglia following hypoxia treatment, BV-2 cells were incubated at a density of 1 × 10^6^ cells in 60 mm culture dishes overnight. Then, BV2 microglia were subjected to hypoxia (1% O_2_) for 12 h in the absence or presence of dexmedetomidine as described in the preceding paragraph. The Control group received saline as the vehicle. The supernatants of BV2 microglia (termed CM) were harvested, filtered, and directly added to primary hippocampal neurons, and incubation continued for 24 h.

### 2.6. Primary Hippocampal Neuronal Cultures

Primary hippocampal neuronal cultures were obtained from P0 SD rat pups as previously described [32]. Briefly, hippocampal tissues were dissected and then digested with 2 mg/ml papain (Macklin Inc., Shanghai, China) at 37°C for 30 min. Single-cell suspensions were obtained by aspiration, filtration, and centrifugation. Cells were seeded on plates precoated with poly-D-lysine at a density of 1 × 10^6^ cells/well for 6-well plates or 1.5 × 10^4^ cells/well for 96-well plates. The cells were incubated in Neurobasal-A medium (Gibco, USA) containing 2% B-27 (Gibco, USA) and 0.5 mM glutamine (HyClone, USA), and half of the medium was changed every 3 days. The primary hippocampal neurons were allowed to differentiate for 7 days before use (Supplemental Figure 1). The purity of the primary hippocampal neurons in the cultures was >90% (Supplemental Figure 2).

### 2.7. Cell Viability and Apoptosis Analysis in Primary Hippocampal Neurons

On day 7, the neuronal medium was removed and replaced with CM from BV2 microglia. Primary hippocampal neurons were incubated with microglia-CM for 24 h, and then, cell viability was assessed using the Cell Counting Kit-8 (CCK-8) assay (Dojindo Molecular Technologies, Kumamoto, Japan). Ten microliters of the CCK-8 solution was added to each well of 96-well plates and incubated at 37°C for 2 h. The absorbance was measured at 450 nm using a microplate reader (Biotek Instruments, Inc., Vermont, USA), and the results are expressed as the percentages of the control level.

The extent of apoptosis was evaluated by flow cytometry using an Annexin V-FITC/PI apoptosis detection kit following the manufacturer's instructions (Beyotime, Shanghai, China). Briefly, hippocampal neurons were collected from 6-well plates and then incubated in 100 *μ*l binding buffer containing 5 *μ*l of PI and 10 *μ*l of Annexin V*-*FITC in the dark at room temperature for 20 min. Apoptosis was analyzed by a flow cytometer (BD Bioscience, San Jose, CA, USA).

### 2.8. Measurement of Intracellular ROS Production in BV2 Microglia

BV2 microglia were cultured at a density of 5 × 10^4^ cells/well in 24-well plates. After the corresponding treatments were administered, intracellular ROS production was measured in BV2 microglia using the *DCFH-DA* fluorescent probe (Beyotime). The microglia were incubated with 10 *μ*M DCFH-DA for 30 min in the dark at 37°C. Fluorescence images were observed under an inverted fluorescence microscope (DMI4000B, Leica, Germany). The fluorescence intensity of images was analyzed by ImageJ software (BX50-FLA, Olympus), and results are expressed as percentages of control levels.

### 2.9. Measurement of Proinflammatory Cytokines in BV2 Microglia by ELISA

BV2 microglia were cultured at a density of 5 × 10^4^ cells/well in 24-well plates. After the corresponding treatment, the concentrations of proinflammatory cytokines including IL-1*β*, IL-6, and TNF-*α* in the supernatants were measured using ELISA kits following the manufacturer's instructions (Proteintech, Wuhan, China). The data are expressed in pg/ml.

### 2.10. Morris Water Maze (MWM) Test

Four weeks after treatment (P31), the MWM test was used to examine the spatial learning and memory ability of rats as previously described [33]. The MWM test was performed for 6 days; it included a 5-day place navigation test and a probe trial on the sixth day. The maze was divided into four quadrants, and a white platform (12 cm in diameter) was submerged in one quadrant of the pool. A video camera above the pool was used to capture the rats' movements for analysis. At P31, before the formal test, the rats were allowed to swim freely in the maze for 90 s to acclimate. At P32–P36, in the place navigation test, the rats were placed facing the wall of the pool and allowed 90 s to find the escape platform. Rats that failed to find the hidden platform in time were guided to the platform, where they were allowed to remain for 30 s. Four trials of the experiment were conducted per day for 5 consecutive days. The average escape latency time was measured to evaluate spatial learning and memory ability. At P37, in the probe trial test, the rats were allowed to swim in the pool without the hidden platform for 90 s. We calculated the number of platform crossings and the time spent in the target quadrant to evaluate spatial memory ability.

### 2.11. Transmission Electron Microscopy (TEM)

Four weeks after neonatal hypoxia, the ultrastructural changes in synapses in the hippocampus and cerebral cortex were evaluated by TEM as described previously [34]. The cerebral tissues were dissected into 1 mm^3^ tissue blocks, and fixed in 2% glutaraldehyde at 4°C for 2 h. The tissue was rinsed in cacodylate buffer, postfixed with 1% osmium tetroxide for 2 h, and then dehydrated in a graded ethanol series. Subsequently, the tissue was infiltrated with a mixture of one-half propylene oxide overnight and embedded in resin. After that, 70 nm sections were stained with 3% uranyl acetate for 20 min and 0.5% lead citrate for 5 min. Five pictures of each subregion per ultrathin section were taken at 9700x or 37000x magnification. The number of synapses, thickness of the postsynaptic density (PSD), and width of the synaptic cleft were measured using ImageJ software (BX50-FLA, Olympus, Japan).

### 2.12. Western Blot Analysis for In Vitro and In Vivo Experiments

The experiments were performed as previously described [28]. The protein concentrations of BV-2 microglia or rat hippocampal tissues were assessed by a BCA protein assay kit (Beyotime). Equal amounts of protein samples (40 *μ*g) were loaded onto a 10% or 12% gradient polyacrylamide gel, transferred to a PVDF membrane, and then incubated overnight at 4°C with primary antibodies against the following proteins: PSD-95 (1 : 1000, Abcam, Cambridge, MA, USA), synaptophysin (1 : 1000, Abcam), NOX2 (1 : 200, Santa Cruz Biotechnology, CA, USA), 4-hydroxynonenal (4-HNE, 1 : 1000, Abcam), IL-1*β* (1 : 500, Cell Signaling Technology, Inc., Massachusetts, USA), IL-6 (1 : 500, Cell Signaling), and TNF-*α* (1 : 500, Cell Signaling). GAPDH (1 : 1000, Cell Signaling) was used as an internal control. After that, the membranes were incubated with horseradish peroxidase-conjugated secondary antibodies (1 : 500, Cell Signaling). The blots were detected using an enhanced chemiluminescence detection system (Pierce Biotechnology, Appleton, WI, USA) with an image analyzer (Bio-Rad Laboratories, CA, USA). Nuclear and cytoplasmic proteins were separately extracted using a nucleoprotein extraction kit (Beyotime). The nuclear protein extracts were subjected to Western blot analysis for NF-*κ*B p65 (1 : 1000, Cell Signaling) and Lamin B1 (1 : 1000, Cell Signaling). The data are expressed as percentages of the control levels.

### 2.13. Immunofluorescence Staining for the In Vitro and In Vivo Experiments

BV2 microglia were cultured at a density of 1 × 10^4^ cells/well in 8-well chamber slides (Millipore, Carrigtwohill, Ireland). After the corresponding treatments were administered, the cells were fixed with 4% paraformaldehyde (PFA) and blocked with 3% bovine serum albumin (BSA) containing 0.5% Triton X-100. The cells were then incubated with anti-NOX2 (1 : 50, Santa Cruz), anti-Iba1 (1 : 100, Wako Pure Chemical Industries., Ltd, Osaka, Japan), or anti-NF-*κ*B p65 (1 : 100, Cell Signaling) primary antibodies overnight at 4°C. After washing with PBS, the cells were incubated with Alexa 546- or Alexa 488-conjugated secondary antibodies (1 : 1000, Life Technologies, Carlsbad, CA, USA) for 1 h. The cell nuclei were counterstained with 0.1 *μ*g/ml DAPI (Beyotime). Fluorescence images were taken using a confocal laser-scanning microscope (FV10i, Olympus, Tokyo, Japan).

Anesthetized rats were transcardially perfused with 4% PFA. Then, the brains were quickly isolated and postfixed in 4% PFA for 24 h and separately dehydrated in 15% and 30% sucrose at 4°C. The brains were embedded in optimum cutting compound and sectioned at a thickness of 20 *μ*m in the coronal plane using a freezing microtome. The sections were blocked with 3% BSA for 1 h at room temperature and then incubated overnight at 4°C with anti-NOX2 (1 : 50, Santa Cruz) and anti-Iba-1 (1 : 100, Wako) primary antibodies. After washing with PBS, the sections were then incubated with Alexa 546- and Alexa 488-conjugated secondary antibodies (1 : 1000, Life Technologies) for 1 h at 37°C. The cell nuclei were counterstained with 0.1 *μ*g/ml DAPI (Beyotime). Fluorescence images were taken using a fluorescence microscope (IX71, Olympus, Tokyo, Japan).

### 2.14. Measurement of NOX Activity for the In Vitro and In Vivo Experiments

NOX activity was evaluated by a cytochrome c reduction assay following the manufacturer's instructions (Genmed Pharmaceutical Technology Co., Ltd., Shanghai, China). Briefly, the supernatants of cell lysates or brain hippocampal tissues were incubated with oxidized cytochrome c in a quartz cuvette for 3 min at 37°C, and then NOX substrate was added to the reaction mixture and incubated for 15 min. Cytochrome c reduction was monitored continuously using a spectrophotometer at 550 nm. NOX activity was estimated by calculating the cytochrome c reduction per min and is expressed in nmol/min/mg protein.

### 2.15. Statistical Analysis

The data were analyzed with SPSS 22.0 software (SPSS Inc., Chicago, USA). All values are expressed as the mean ± standard deviation (SD). In the MWM tests, escape latencies were evaluated by repeated-measures two-way analysis of variance (ANOVA) with “day” as the within-subject factor and “group” as the between-subject factor. Other data were analyzed using one-way ANOVA followed by Bonferroni's post hoc tests. A *P* value < 0.05 was considered statistically significant.

## 3. Results

### 3.1. Dexmedetomidine Attenuated Hypoxia-Induced Cognitive Impairment

**Figure 1:**
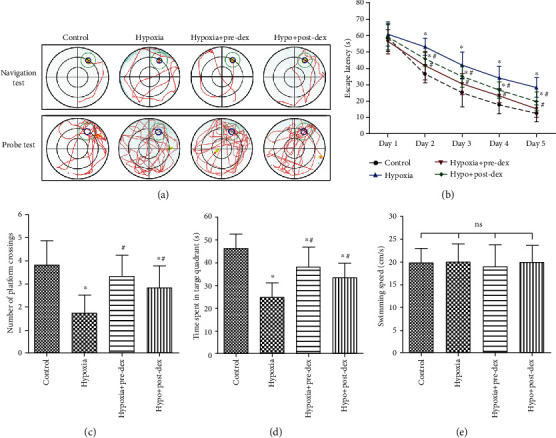
Dexmedetomidine ameliorated hypoxia-induced cognitive impairment in juvenile rats. Neonatal rats were treated with dexmedetomidine 30 min before or immediately after hypoxia exposure, and spatial learning and memory ability were measured with the MWM test 28 days after hypoxic insult. (a) Representative swimming tracks (red routes) of the rats from the place navigation test on day 5 and the probe test on day 6. (b) Average escape latency to reach the hidden platform during the place navigation test over days 1 to 5. (c) Number of platform crossings during the probe test. (d) Time spent in the target quadrant during the probe test. (e) Swimming speeds of the rats in different groups during the probe test. The data are expressed as the mean ± SD, *n* = 12. ^∗^*P* < 0.05 vs. the Control group, ^#^*P* < 0.05 vs. the Hypoxia group. Hypo: hypoxia; Pre-Dex: dexmedetomidine pretreatment; Post-Dex: dexmedetomidine posttreatment.

We first evaluated the effects of dexmedetomidine on spatial learning and memory ability in hypoxic neonatal rats by using the MWM test. As shown in Figure 1(b), all groups showed rapid decreases in escape latency over the 5 days of the navigation test (*P* < 0.05), and no significant interaction was found between training days and groups (*P* > 0.05), indicating an improvement in spatial learning and memory over time, regardless of previous treatment. There were no significant differences in escape latency among the groups on day 1 (*P* > 0.05). However, the escape latency in the Hypoxia group was much longer than that in the Control group from days 2 to 5. Interestingly, both pre- and posttreatment with dexmedetomidine notably shortened the escape latency from days 2 to 5 compared to that of the Hypoxia group, indicating cognitive improvement.

In the probe trial on day 6, hypoxia-exposed rats displayed significantly fewer platform crossings (*P* < 0.05; Figure 1(c)) and less time spent in the target quadrant than the controls (*P* < 0.05; Figure 1(d)), indicating memory impairment. However, the number of platform crossings (*P* < 0.05) and the time spent in the target quadrant (*P* < 0.05) were significantly higher in both the Hypo+Pre-Dex and Hypo+Post-Dex groups compared with the Hypoxia group. The protective effects tended to be more pronounced in the Hypo+Pre-Dex group than in the Hypo+Post-Dex, although they were not statistically different between the groups. In addition, swimming speed did not differ between the groups (*P* > 0.05; Figure 1(e)), indicating that the observed differences in the MWM test outcomes were due to changes in cognitive function.

### 3.2. Dexmedetomidine Attenuated Synaptic Loss after Neonatal Hypoxia

**Figure 2:**
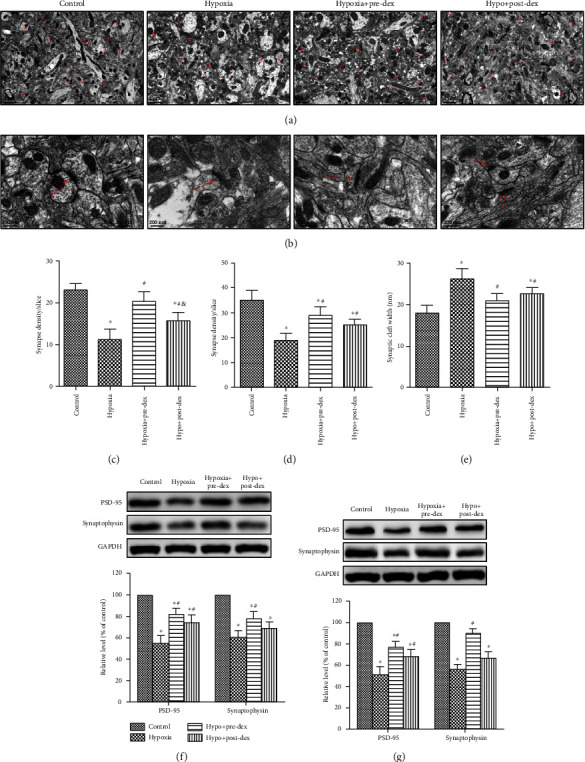
Dexmedetomidine attenuated hypoxia-induced synaptic loss in the hippocampus. Neonatal rats were treated with dexmedetomidine 30 min before or immediately after hypoxia exposure, and (a–e) synaptic ultrastructure changes in the hippocampal CA1 region were observed under TEM 28 days following hypoxia. (a) Representative photomicrograph (9700x) showing the differences in the number of synapses per slice among the four groups (the red arrows indicate the synapses). (b) Representative high-magnification photomicrograph (37000x) showing the differences in the thickness of PSD and the width of the synaptic cleft among the four groups (the red arrows indicate the synaptic linkage). (c–e) Quantification of synapse density, PSD thickness and synaptic cleft width from at least 20 sections among the four groups. The protein expression of PSD95 and synaptophysin was measured in the hippocampus 24 h (f) and 28 days (g) after hypoxic insult by Western blot analysis. The levels of PSD-95 and synaptophysin expression are presented as the percentages of those in the Control group. The data are expressed as the mean ± SD, *n* = 5. ^∗^*P* < 0.05 vs. the Control group, ^#^*P* < 0.05 vs. the Hypoxia group, ^&^*P* < 0.05 vs. the Hypo+Pre-Dex group. Hypo: hypoxia; Pre-Dex: dexmedetomidine pretreatment; Post-Dex: dexmedetomidine posttreatment.

Synaptic loss or decreased synaptic density is believed to be closely linked to cognitive impairment [35]. We next evaluated the effects of dexmedetomidine on synaptic loss. We first examined synaptic density and ultrastructure in the hippocampus using TEM 28 days after neonatal hypoxia. We observed that the number of synapses in the hippocampal CA1 region was remarkably lower posthypoxic insult than under control conditions (*P* < 0.05; Figures 2(a) and 2(c)). Moreover, the synaptic cleft was widened, and the PSD thickness was dramatically reduced in the Hypoxia group compared with the Control group (*P* < 0.05; Figures 2(b), 2(d), and 2(e)). In contrast, these impairments in the hippocampal synaptic ultrastructure were improved in the Hypo+Pre-Dex and Hypo+Post-Dex groups compared to the Hypoxia group (both *P* < 0.05), with dexmedetomidine pretreatment proving to be slightly more effective than dexmedetomidine posttreatment (*P* < 0.05; Figure 2(c)).

Subsequent Western blot analysis showed that the postsynaptic protein PSD-95 and the presynaptic protein synaptophysin were substantially downregulated in the hippocampus 1 day and 28 days after hypoxic insult (both *P* < 0.05, Figures 2(f) and 2(g)). Similar to the TEM results, both pre- and posttreatment with dexmedetomidine efficiently reversed the hypoxia-induced decrease in PSD-95 expression (*P* < 0.05). Interestingly, compared with that in the Hypoxia group, the expression level of synaptophysin was obviously higher in the Hypo+Pre-Dex group (*P* < 0.05) but not in the Hypo+Post-Dex group (*P* > 0.05) at 1 day and 28 days after hypoxic insult. Our results indicated that neonatal hypoxia resulted in hippocampal synaptic loss during the early and advanced stages of development. Importantly, dexmedetomidine significantly reversed these impairments under hypoxic conditions. In contrast to the severity of synaptic impairment in the hippocampus, neonatal hypoxia resulted in relatively mild synaptic loss in the cerebral cortex, as evidenced by less damaged synapses and higher expression levels of synaptophysin and PSD-95 than that seen in the hippocampus following hypoxic insult. As expected, dexmedetomidine treatment also partly improved hypoxia-induced synaptic impairment in the cortex (Supplemental Figure 3).

### 3.3. Dexmedetomidine Suppressed Hypoxia-Induced Microglial NOX2 Activation In Vivo and In Vitro

**Figure 3:**
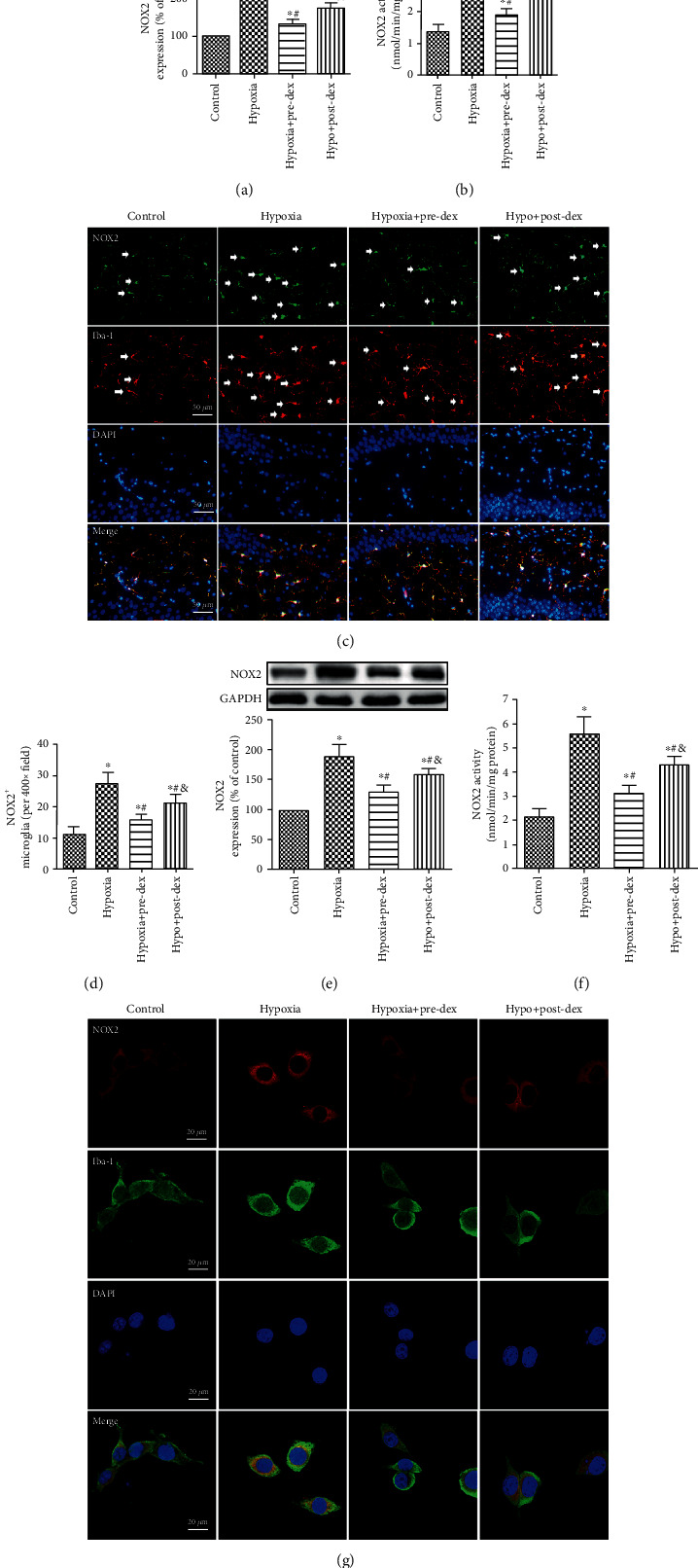
Dexmedetomidine suppressed NOX2 activation in hippocampal microglia and cultured BV2 microglia following hypoxia. (a–d) Neonatal rats were treated with dexmedetomidine 30 min before or immediately after hypoxia exposure. (a) Representative Western blot images of NOX2 in the hippocampus were taken 24 h after neonatal hypoxia, and the level of NOX2 expression is presented as the percentage of that in the Control group. (b) NOX2 activity was measured using a cytochrome c reduction assay. (c) Immunofluorescence of NOX2 (green), microglia (red, labeled by Iba-1), and DAPI (blue) in the hippocampal CA1 region (the white arrowheads show NOX2 and Iba-1 colabeled cells). (d) Number of NOX2^+^ microglia in the hippocampal CA1 region. (e–g) Microglia were treated with dexmedetomidine 2 h before or immediately after hypoxia exposure. (e) Representative Western blot images of NOX2 in cultured BV2 microglia harvested 24 h after hypoxia exposure, and the level of NOX2 expression is presented as the percentage of that in the Control group. (f) NOX2 activity was measured using a cytochrome c reduction assay. (g) Immunofluorescence of NOX2 (red), microglia (green), and DAPI (blue) in BV2 cells. Representative photomicrographs were captured under a confocal microscope. The data are expressed as the mean ± SD, *n* = 5. ^∗^*P* < 0.05 vs. the Control group, ^#^*P* < 0.05 vs. the Hypoxia group, ^&^*P* < 0.05 vs. the Hypo+Pre-Dex group. Hypo: hypoxia; Pre-Dex: dexmedetomidine pretreatment; Post-Dex: dexmedetomidine posttreatment.

To determine the extent of NOX2 activation in hypoxic microglia, we examined the expression and activity of NOX2 in the developing hippocampus and in cultured BV2 microglia. We found that NOX2 protein expression and activity were both obviously increased in the hippocampus 1 day after hypoxic insult (*P* < 0.05; Figures 3(a) and 3(b)). However, these increases could largely be reversed by pre- and posttreatment with dexmedetomidine (both *P* < 0.05). It has been previously reported that NOX2 is highly expressed in activated microglia following acute brain damage [17]. Similarly, we observed that hypoxia exposure obviously increased the number of NOX2^+^ cells, which were almost fully colabeled with Iba-1 (*P* < 0.05; Figures 3(c) and 3(d)). Both pre- and posttreatment with dexmedetomidine reduced the number of these cells in the hippocampus, with pretreatment with dexmedetomidine proving to be more effective (*P* < 0.05).

Consistent with the above *in vivo* data, our *in vitro* results revealed that hypoxia exposure significantly upregulated the expression and activity of NOX2 in BV2 microglia; however, the increases were attenuated in both the Hypo+Pre-Dex and Hypo+Post-Dex groups (all *P* < 0.05; Figures 3(e) and 3(f)). Moreover, dual immunofluorescence analysis of Iba-1 and NOX2 in BV2 microglia was performed. As shown in Figure 3(g), the BV2 microglia in the Control group exhibited small somas with long distal arborizations, as well as relatively weak fluorescence intensity of NOX2. In contrast, hypoxia-exposed BV2 microglia were larger and round with strong NOX2 fluorescence intensity. However, both pre- and posttreatment with dexmedetomidine attenuated the hypoxia-induced changes in morphology and NOX2 fluorescence intensity in BV2 microglia. These results demonstrated that dexmedetomidine significantly suppressed hypoxia-induced NOX2 activation in microglia.

### 3.4. Dexmedetomidine Attenuated Hypoxia-Induced Oxidative Stress In Vivo and In Vitro

**Figure 4:**
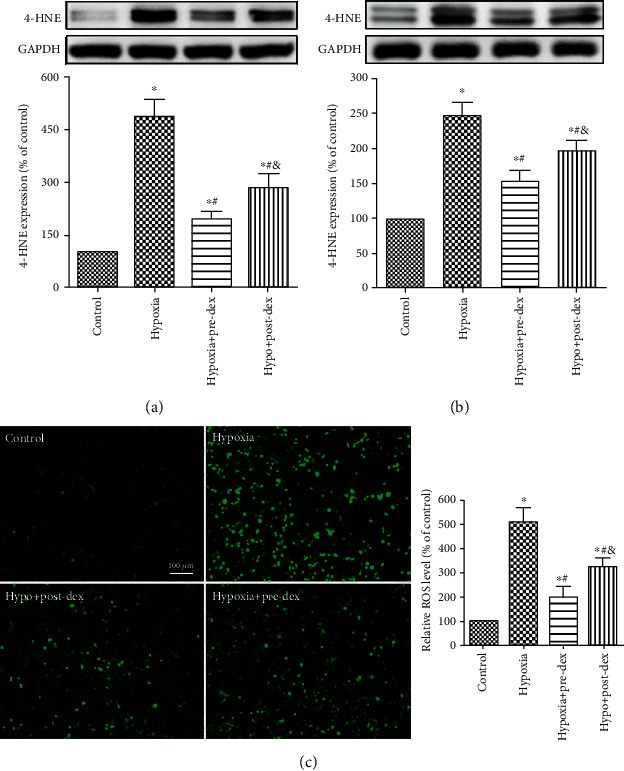
Dexmedetomidine attenuated hypoxia-induced oxidative stress in the developing hippocampus and in cultured BV2 microglia. (a) Neonatal rats were treated with dexmedetomidine 30 min before or immediately after hypoxia exposure, and the expression of 4-HNE was measured in the hippocampus 24 h after hypoxia exposure by Western blot analysis. (b) Microglia were treated with dexmedetomidine 2 h before or immediately after hypoxia exposure, and the expression of 4-HNE was measured in BV2 cells 24 h after hypoxia by Western blot analysis. The levels of 4-HNE expression are presented as the percentage of that in the Control group. (c) Intracellular ROS production was measured with the DCHF-DA probe, and the relative fluorescence intensity was determined by ImageJ software. The ROS levels are presented as the percentage of that in the Control group (mean ± SD, *n* = 4). ^∗^*P* < 0.05 vs. the Control group, ^#^*P* < 0.05 vs. the Hypoxia group, ^&^*P* < 0.05 vs. the Hypo+Pre-Dex group. Hypo: hypoxia; Pre-Dex: dexmedetomidine pretreatment; Post-Dex: dexmedetomidine posttreatment.

In microglia, ROS originate mainly from NOX2, and the activation of microglial NOX2 is related to excessive oxidative stress and inflammation, which may result in synaptic loss and cognitive impairment [15]. 4-HNE is a major product of lipid peroxidation and is widely used as a reliable marker of increased oxidative stress. As expected, 4-HNE protein expression was increased in both the developing hippocampus and in BV2 microglia 1 day after hypoxic insult (*P* < 0.05; Figures 4(a) and 4(b)). Both the Hypo+Pre-Dex and Hypo+Post-Dex groups exhibited obviously lower expression of 4-HNE than the Hypoxia group (*P* < 0.05).

Consistent with the Western blot results, exposure to hypoxia for 12 h significantly increased ROS levels in BV2 microglia, and dexmedetomidine pre- and posttreatment effectively attenuated this increase, with dexmedetomidine pretreatment proving to be more effective than posttreatment (*P* < 0.05; Figure 4(c)).

### 3.5. Dexmedetomidine Suppressed Hypoxia-Activated NF-*κ*B Signaling and Proinflammatory Cytokine Production In Vivo and In Vitro

**Figure 5:**
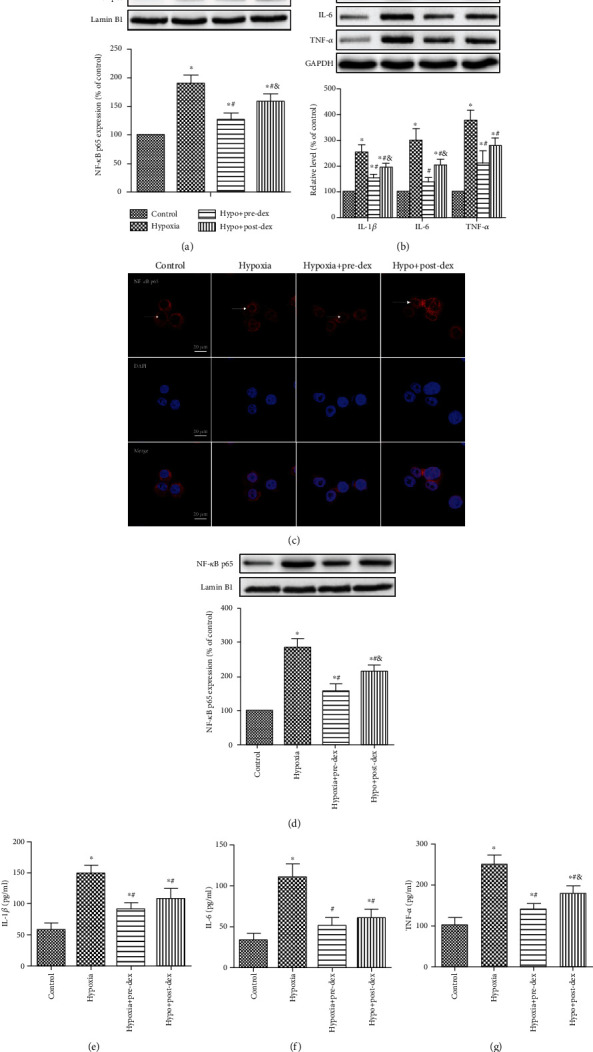
Dexmedetomidine suppressed NF-*κ*B activation and proinflammatory cytokine production in the developing hippocampus and in cultured BV2 microglia following hypoxia. (a, b) Neonatal rats were treated with dexmedetomidine 30 min before or immediately after hypoxia exposure. Then, the expression of nuclear NF-*κ*B p65 and proinflammatory cytokines, including IL-1*β*, IL-6, and TNF-*α*, in the hippocampus was measured 24 h after neonatal hypoxia by Western blot analysis. Representative Western blot images and densitometric analyses are presented. (c–g) Microglia were treated with dexmedetomidine 2 h before or immediately after hypoxia exposure, and (c) representative immunofluorescent images of NF-*κ*B p65 (red) and DAPI (blue) in BV2 microglia 24 h after hypoxia exposure were captured under a confocal microscope. (d) The expression of nuclear NF-*κ*B p65 was measured in BV2 cells by Western blot analysis and is presented as the percentage of that in the Control group. (e–g) The levels of IL-1*β*, IL-6, and TNF-*α* in the cell supernatants of cultured BV2 microglia were determined with ELISA kits. The data are represented as percentages of the values in the Control group (mean ± SD, *n* = 4). ^∗^*P* < 0.05 vs. the Control group, ^#^*P* < 0.05 vs. the Hypoxia group, ^&^*P* < 0.05 vs. the Hypo+Pre-Dex group. Hypo: hypoxia; Pre-Dex: dexmedetomidine pretreatment; Post-Dex: dexmedetomidine posttreatment.

NOX2-derived ROS have been reported to be involved in neurotoxic microglial activation because of their regulation of NF-*κ*B signaling [18]. To determine whether NOX2 is involved in neonatal hypoxia-activated NF-*κ*B signaling, the levels of nuclear NF-*κ*B p65 in the developing hippocampus and in BV2 microglia were first examined. Our results indicated that the NF-*κ*B signaling pathway was activated following hypoxia exposure, as evidenced by increased nuclear protein levels of NF-*κ*B p65 in the Western blot analysis (both *P* < 0.05, Figures 5(a) and 5(d)) and by nuclear translocation of p65 NF-*κ*B revealed by immunofluorescence (Figure 5(c)). Importantly, both pre- and posttreatment with dexmedetomidine were effective in suppressing hypoxia-activated NF-*κ*B signaling in the hippocampus and BV2 microglia, with dexmedetomidine pretreatment proving to be more effective than posttreatment (both *P* < 0.05).

It is well known that NF-*κ*B is a key upstream regulator of the production of proinflammatory mediators. Therefore, we examined the levels of several key proinflammatory cytokines (IL-1*β*, IL-6, and TNF-*α*) in the neonatal rat hippocampus and BV2 microglial supernatant. As expected, the levels of IL-1*β*, IL-6, and TNF-*α* were substantially increased in developing hippocampus and BV2 microglia supernatant 1 day after hypoxic insult (*P* < 0.05, Figure 5(b); *P* < 0.05, Figures 5(e)–5(g)), while both pre- and posttreatment with dexmedetomidine efficiently reduced the levels of these molecules (*P* < 0.05). These results suggested that dexmedetomidine exhibited potent anti-inflammatory activity against hypoxia-induced neuroinflammation.

### 3.6. Dexmedetomidine-Mediated Attenuation of Hypoxia-Induced Synaptic Loss Was Related to Modulation of NOX2 Activation in the Hippocampus

**Figure 6:**
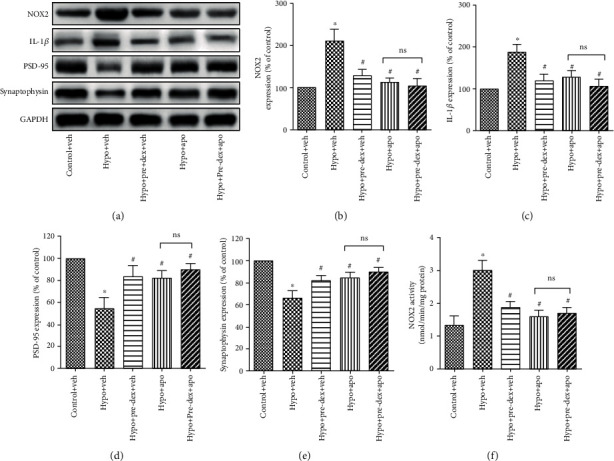
The NOX inhibitor apocynin protected against hypoxia-induced proinflammatory cytokine production and synaptic loss in the developing hippocampus. Neonatal rats were pretreated with dexmedetomidine or apocynin before hypoxia exposure, and then the expression of NOX2, IL-1*β*, PSD-95, and synaptophysin in the hippocampus was measured at 24 h after neonatal hypoxia by Western blot analysis. Representative Western blot images (a) and densitometric analyses (b–e) are presented. (f) NOX2 activity was measured using a cytochrome c reduction assay. The data are expressed as the mean ± SD, *n* = 4. ^∗^*P* < 0.05 vs. the Control+Veh group, ^#^*P* < 0.05 vs. the Hypo+Veh group. ns: no significance; Hypo: hypoxia; Pre-Dex: dexmedetomidine pretreatment; Post-Dex: dexmedetomidine posttreatment; Apo: apocynin; Veh: vehicle.

Next, to specifically confirm whether the protective effect of dexmedetomidine against hypoxia-induced synaptic loss was attributed to its regulation of NOX2 activation, we used the NOX-specific inhibitor apocynin to evaluate the role of NOX2 in this process. As shown in Figure 6, pretreatment with apocynin significantly downregulated NOX2 and IL-1*β* protein expression and NOX2 activity while simultaneously upregulating the protein expression of PSD-95 and synaptophysin in the hippocampus compared with the expression in the vehicle-pretreated Hypoxia group (Hypo+Veh group) (*P* < 0.05). Moreover, the combination of dexmedetomidine and apocynin did not significantly change the expression levels of the synaptic proteins and IL-1*β* from those observed in the vehicle-pretreated Hypo+Pre-Dex group (Hypo+Pre-Dex + Veh group) (*P* > 0.05), suggesting that the neuroprotective function of dexmedetomidine against hypoxia-induced synaptic loss was strongly linked to inhibition of NOX2 activation.

### 3.7. Dexmedetomidine-Mediated Protection against Hypoxia-Induced Neuronal Damage Was Related to Regulation of NOX2 in Microglia

**Figure 7:**
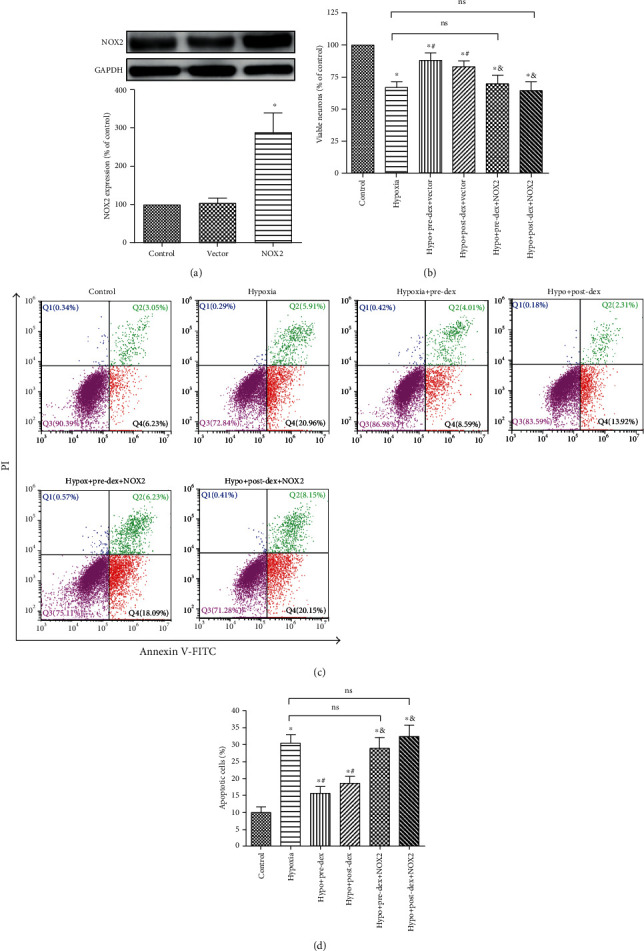
Overexpression of NOX2 in BV2 microglia eliminated the protective effect of dexmedetomidine against hypoxia-induced neuronal damage. (a) The infection efficiency of the control lentivirus (vector) and NOX2 overexpression lentivirus were measured by Western blot analysis. NOX2 expression is presented as the percentage of that in the Control group. Primary hippocampal neurons were treated with microglia-CM for 24 h; thereafter, (b) cell viability was detected by CCK-8 assay, and (c, d) neuronal apoptosis was measured by Annexin V/PI staining and flow cytometry. Representative images (c) and graphical quantification (d) of apoptosis are presented. The data are expressed as the mean ± SD, *n* = 4. ^∗^*P* < 0.05 vs. the Control group, ^#^*P* < 0.05 vs. the Hypoxia group, ^&^*P* < 0.05 vs. the Hypo+Pre-Dex+Vector or Hypo+Post-Dex+Vector group. ns: no significance; Hypo: hypoxia; Pre-Dex: dexmedetomidine pretreatment; Post-Dex: dexmedetomidine posttreatment.

Finally, to explore the potential role of microglial NOX2 in the dexmedetomidine-induced alleviation of hypoxic neuronal damage, NOX2-overexpressing and control BV2 microglia were exposed to hypoxia with or without dexmedetomidine treatment. After that, primary hippocampal neurons were exposed to microglial-CM for 24 h, and then, cell viability and apoptosis were analyzed. As shown in Figure 7(b), cell viability was significantly lower among neurons exposed to CM from hypoxia-treated microglia than among neurons exposed to CM from normoxia cultured microglia (*P* < 0.05). However, when hippocampal neurons were incubated in CM from hypoxic microglia pre- and posttreatment with dexmedetomidine, cell viability was significantly increased (*P* < 0.05). Interestingly, overexpression of NOX2 in microglia effectively inhibited the dexmedetomidine-mediated improvement in neuronal viability (*P* < 0.05). Flow cytometry analysis of apoptosis further revealed that pre- and posttreatment with dexmedetomidine significantly prevented hippocampal neuronal apoptosis induced by hypoxic microglia-CM (*P* < 0.05; Figures 7(c) and 7(d)). Furthermore, overexpression of NOX2 effectively inhibited the dexmedetomidine-mediated improvement in neuronal apoptosis (*P* < 0.05). Overall, these results indicated that dexmedetomidine attenuated hypoxia-induced neurotoxicity and was closely related to the inhibition of microglial NOX2.

## 4. Discussion

We have previously demonstrated that dexmedetomidine pretreatment can provide neuroprotection against hypoxia-induced oxidative damage in neuron-like PC12 cells [28]. On the basis of these findings, the present study further examined whether dexmedetomidine could ameliorate synaptic and cognitive impairment and explored the potential mechanisms in hypoxic neonatal rats. Our main findings include the following: (1) both pre- and posttreatment with dexmedetomidine alleviated hypoxia-induced cognitive impairment and synaptic loss and restored the expression of two dominant synaptic proteins (PSD95 and synaptophysin) in the hippocampus; (2) the neuroprotective benefits of dexmedetomidine in neonatal rats and cultured hippocampal neurons were linked to suppression of the neuroinflammatory response and attenuation of oxidative stress; (3) dexmedetomidine treatment prevented NOX2-mediated synaptic loss and neuronal damage; (4) blocking microglial NOX2 activation might contribute to the anti-inflammatory and antioxidant effects of dexmedetomidine in the developing hippocampus following hypoxic insult.

Numerous studies have demonstrated that neuronal synaptic plasticity, which involves changes in synapse number, ultrastructure, and proteins, is the neurobiological basis of memory and cognitive function [11]. In our study, the learning and memory deficits observed in hypoxia-exposed neonatal rats were linked to synaptic damage and decreased levels of synaptic proteins. Consistent with our findings, previous studies have also shown that the number of synapses and the expression of synaptic proteins are reduced in a neonatal hypoxia-ischemia mouse model [34]. More importantly, we found for the first time that dexmedetomidine pre- and posttreatment both effectively ameliorated hypoxia-induced cognitive impairment and hippocampal synaptic loss, as evidenced by significant increases in PSD95 expression, synapse numbers, and PSD thickness during early and advanced stages of postnatal development. Synaptic loss in the hippocampus has long been considered an early and vital pathophysiological hallmark of neurodegenerative disorders [35, 36]. Thus, an additional mechanism by which dexmedetomidine treatment improves cognitive function is likely linked to maintenance of synaptic integrity following hypoxic insult.

Acute exposure to low ambient oxygen levels has been demonstrated to cause mild to moderate cell damage events in the developing brain. Similarly, in our study, acute systemic hypoxia in 3-day-old rats resulted in synaptic and cognitive impairment. In contrast to the severity of synaptic impairment in the hippocampus, neonatal hypoxia resulted in relatively mild synaptic loss in the cerebral cortex. Our findings are in agreement with the selective vulnerability observed after hypoxic insult *in vivo* and *in vitro*, showing that the hippocampus, a well-studied structure associated with memory and cognitive function, is the most vulnerable brain region following neonatal hypoxia [10, 37, 38]. During the process of reoxygenation, secondary inflammation, oxidative stress, and excitotoxicity collectively contribute to neuronal and synaptic damage and can persist for days to weeks [29, 39, 40]. Based on the above findings, we next deeply explored the underlying mechanisms and molecular effects of dexmedetomidine in the regulation of hippocampal synaptic plasticity in hypoxic neonatal rats.

Microglia are the principal immune cells in the central nervous system (CNS) and can interact specifically with neighboring neurons once activated [18]. Previous studies have indicated that dexmedetomidine posttreatment protected against hypoxic-ischemic brain damage, possibly through inhibition of neuroinflammation in neonatal rats, but how it does so remains unclear. NOX2 activity has recently emerged as a common and essential mechanism underlying microglia-mediated neurotoxicity [18, 20]. Specifically, the NOX2 protein is located mainly at the microglial cell membrane, where ROS produced outside of the cell can directly damage neurons through an extracellular pathway [17]. It has been reported that hypoxia triggers Ca^2+^ entry from extracellular sources through voltage-gated calcium channels (VGCC), and increased levels of intracellular Ca^2+^ play a key role in regulating NOX2 activation in the CNS [41–43]. We demonstrated here that NOX2 expression and activity were significantly increased in the developing hippocampus and cultured microglia after hypoxic insult. Notably, pre- and posttreatment with dexmedetomidine markedly suppressed microglial NOX2 activation and attenuated subsequent oxidative stress following hypoxia *in vivo* and *in vitro*. Previous studies from our laboratory and others have indicated that dexmedetomidine potently suppresses overload Ca^2+^ entry through VGCC and NMDA receptors in the rats hippocampus and neuron-like PC12 cells following hypoxia [28, 44, 45]. Therefore, we could speculate that modulation of intracellular Ca^2+^ levels may contribute to dexmedetomidine-mediated NOX2 activation in hypoxic brain damage in neonates. Although ROS are favorable for synaptic function under physiological conditions, oxidative stress in synapses has been implicated in the pathology of several neurodegenerative disorders, including Alzheimer's disease, cerebral ischemia, and TBI [14, 46]. Accordingly, we proposed that dexmedetomidine exerts neuroprotection partially via inhibition of microglial NOX2-derived oxidative stress under hypoxic conditions.

There is a close interdependence between oxidative stress and neuroinflammation. Increases in intracellular ROS are known to promote NF-*κ*B activation, which subsequently regulates the transcription of proinflammatory cytokines and other mediators involved in acute inflammation [47, 48]. Dexmedetomidine has been reported to prevent NF-*κ*B translocation into the nucleus and thus reduce the production of proinflammatory cytokines, thus exerting a protective effect against brain damage [49, 50]. Consistent with these findings, our data showed that dexmedetomidine pre- and posttreatment both effectively attenuated NF-*κ*B activation and inflammatory cytokine production in the developing hippocampus and cultured microglia subjected to hypoxia.

To specifically confirm whether the protective effects of dexmedetomidine against neonatal hypoxia-induced neuroinflammation and synaptic impairment were attributable to its regulation of microglial NOX2 activation, we further used the NOX inhibitor apocynin *in vivo* and lentivirus-mediated NOX2 overexpression *in vitro* to evaluate the role of NOX2 in this process. As expected, pretreatment with apocynin abolished the enhanced NOX2 protein expression and activity and the proinflammatory response in the hippocampus and reversed neonatal hypoxia-induced synaptic loss. In addition, treating primary hippocampal neurons with CM from hypoxia-activated microglia induced neuronal damage, an effect that was reversed by CM from dexmedetomidine-treated microglia with normal NOX2 expression, but not by CM from dexmedetomidine-treated microglia with NOX2-overexpression. Importantly, the effective dose of dexmedetomidine (1 *μ*M) used *in vitro* is clinically relevant, since it is within the range of therapeutic human plasma concentrations [51]. Taken together, these data suggest that pharmacological inhibition of NOX2-derived ROS in hippocampal microglia likely contributed to the alleviation of neuroinflammation and synaptic impairment and is thus a promising potential neuroprotective therapy for hypoxic brain injury in neonates.

In our study, dexmedetomidine applied before or after the neonatal hypoxic phase provided neuroprotection against synaptic and cognitive impairment, with dexmedetomidine pretreatment proving to be more effective than posttreatment. However, considering that most cases of perioperative hypoxia are likely not predicted, the application of dexmedetomidine after the onset of hypoxic insult is more clinically relevant than prehypoxia application. We speculate that the superiority of prehypoxia dexmedetomidine treatment over postreoxygenation dexmedetomidine treatment in this study might be attributable to the slow onset of dexmedetomidine, which reaches its effect approximately 15 min after intravenous administration [23]. Considering the characteristic feature of rapid activation of microglia in response to hypoxic insult, pretreatment of dexmedetomidine 30 min before the onset of neonatal hypoxia could exert a more prominent effect on microglia priming compared with postreatment, resulting in lower levels of oxidative stress and neuroinflammation [52]. To our knowledge, the optimal timing of dexmedetomidine application remains controversial. Wang et al. reported that dexmedetomidine applied immediately or 2 h after the onset of subarachnoid hemorrhage provided neuroprotection in rats [30]. In contrast, a previous study showed that dexmedetomidine applied before ischemia but not after ischemia attenuated intestinal injury induced by intestinal ischemia/reperfusion in rats [53]. At the very least, our results indicate that early application of dexmedetomidine is important for neuronal synaptic protection. This finding may aid clinicians in developing best practices for the use of dexmedetomidine.

There were several limitations in our study. First, the dose of dexmedetomidine (25 *μ*g/kg) in our *in vivo* study was determined according to previous neuroprotection research. Additional doses should be tested in the future to investigate the dose-effect relationship and select an optimal dose that protects against neonatal hypoxic brain damage. Next, no survival benefit of dexmedetomidine was found in our study. A larger sample size might be needed to detect a difference in survival outcomes. Third, we specifically focused on the role of NOX2 in dexmedetomidine efficacy. It is not yet known whether other subtypes of NOX are related to the effects of dexmedetomidine. Forth, hippocampal neurogenesis is considered a critical aspect for the development of learning and memory throughout life [54]. It is not known whether dexmedetomidine could also act through NOX2 to improve neurogensis following neonatal hypoxia; this is a possible future work for our research.

**Figure 8:**
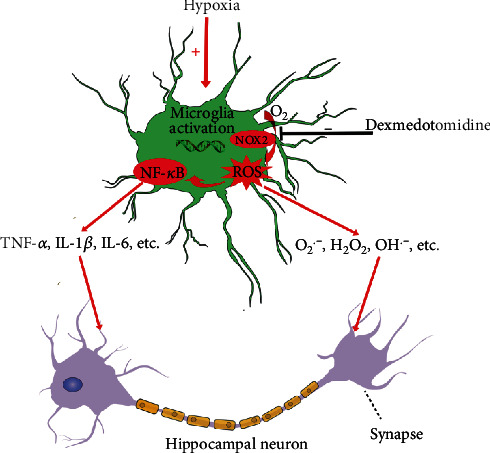
Schematic showing the potential mechanism of the neuroprotective effects of dexmedetomidine against hippocampal synaptic damage under hypoxic conditions. Under hypoxic conditions, microglial NOX2 activation and subsequent ROS generation are key upstream regulators that can activate NF-*κ*B signaling and amplify the production of proinflammatory cytokines. Dexmedetomidine suppresses microglial NOX2 to reduce ROS and neuroinflammation and finally protects against hippocampal synaptic damage.

In conclusion, our study demonstrates that dexmedetomidine alleviates hippocampal synaptic loss and cognitive impairment in rats neonatally exposed to hypoxic insult by suppressing oxidative stress and neuroinflammation through inhibition of microglial NOX2 activation. Hence, according to our data, microglial NOX2 could serve as a promising and effective candidate target for neuroprotection against hypoxic brain damage in neonates (Figure 8).

## Data Availability

The data used to support the findings of this study are available from the corresponding author upon request.

## Supplementary Materials

Supplementary Materials Supplementary materials Figure 1: morphological changes in primary hippocampal neurons at different times. Neuronal morphological changes were observed under an inverted phase-contrast microscope (200x). (a) Neuronal morphology 1 day after cell inoculation, (b) 3 days after cell inoculation, (c) 5-6 days after cell inoculation, and (d) 7-8 days after cell inoculation. Figure 2: the purity of hippocampal neurons was identified by neuron-specific anti-NeuN antibody and DAPI double staining. Representative photomicrographs were captured under a confocal microscope (400x). Figure 3: dexmedetomidine partially attenuated hypoxia-induced synaptic loss in the cerebral cortex. Neonatal rats were treated with dexmedetomidine 30 min before or immediately after hypoxia exposure, and (a–e) synaptic ultrastructure changes in the prefrontal cortex were observed under TEM 28 days following hypoxia. (a) Representative photomicrograph (9700x) showing the differences in the number of synapses per slice among the four groups (the red arrows indicate the synapses). (b) Representative high-magnification photomicrograph (37000x) showing the differences in the thickness of PSD and the width of the synaptic cleft among the four groups (the red arrows indicate the synaptic linkage). (c–e) Quantification of synapse density, PSD thickness and synaptic cleft width from at least 20 sections among the four groups. The protein expression of PSD95 and synaptophysin was measured in the prefrontal cortex 28 days (f, g) after hypoxic insult by Western blot analysis. The levels of PSD-95 and synaptophysin expression are presented as the percentages of those in the Control group. The data are expressed as the mean ± SD, *n* = 4. ^∗^*P* < 0.05 vs. the Control group, ^#^*P* < 0.05 vs. the Hypoxia group. Hypo: hypoxia; Pre-Dex: dexmedetomidine pretreatment; Post-Dex: dexmedetomidine posttreatment.
