# Supplementary material for: Dexmedetomidine Alleviates Hypoxia-Induced Synaptic Loss and Cognitive Impairment via Inhibition of Microglial NOX2 Activation in the Hippocampus of Neonatal Rats
Source: Oxid Med Cell Longev. 2021 Feb 12;2021:6643171. doi: 10.1155/2021/6643171 (PMC7895593; doi:10.1155/2021/6643171)
Supplement: Supplementary Materials — Figure 1: morphological changes in primary hippocampal neurons at different times. Neuronal morphological changes were observed under an inverted phase-contrast microscope (200x). (a) Neuronal morphology 1 day after cell inoculation, (b) 3 days after cell inoculation, (c) 5-6 days after cell inoculation, and (d) 7-8 days after cell inoculation. Figure 2: the purity of hippocampal neurons was identified by neuron-specific anti-NeuN antibody and DAPI double staining. Representative photomicrographs were captured under a confocal microscope (400x). Figure 3: dexmedetomidine partially attenuated hypoxia-induced synaptic loss in the cerebral cortex. Neonatal rats were treated with dexmedetomidine 30 min before or immediately after hypoxia exposure, and (a–e) synaptic ultrastructure changes in the prefrontal cortex were observed under TEM 28 days following hypoxia. (a) Representative photomicrograph (9700x) showing the differences in the number of synapses per slice among the four groups (the red arrows indicate the synapses). (b) Representative high-magnification photomicrograph (37000x) showing the differences in the thickness of PSD and the width of the synaptic cleft among the four groups (the red arrows indicate the synaptic linkage). (c–e) Quantification of synapse density, PSD thickness and synaptic cleft width from at least 20 sections among the four groups. The protein expression of PSD95 and synaptophysin was measured in the prefrontal cortex 28 days (f, g) after hypoxic insult by Western blot analysis. The levels of PSD-95 and synaptophysin expression are presented as the percentages of those in the Control group. The data are expressed as the mean ± SD, n = 4. ∗P < 0.05 vs. the Control group, #P < 0.05 vs. the Hypoxia group. Hypo: hypoxia; Pre-Dex: dexmedetomidine pretreatment; Post-Dex: dexmedetomidine posttreatment. [file 6643171.f1.docx]

**Supplementary data**

**
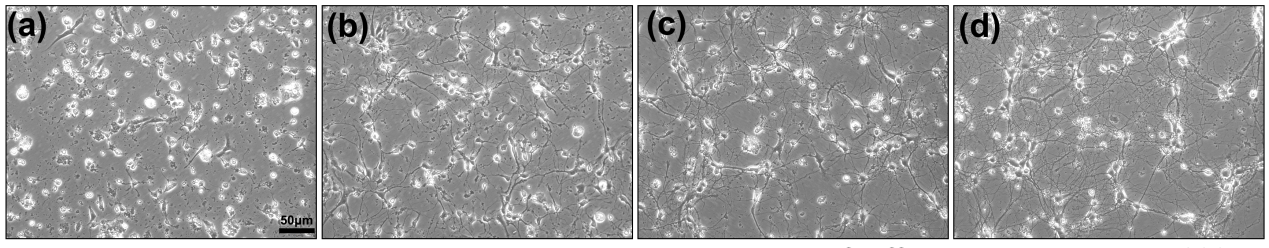
**

Figure 1: Morphological changes in primary hippocampal neurons at different times. Neuronal morphological changes were observed under an inverted phase-contrast microscope (200×). (a) Neuronal morphology 1 day after cell inoculation, (b) 3 days after cell inoculation, (c) 5-6 days after cell inoculation, and (d) 7-8 days after cell inoculation.

**
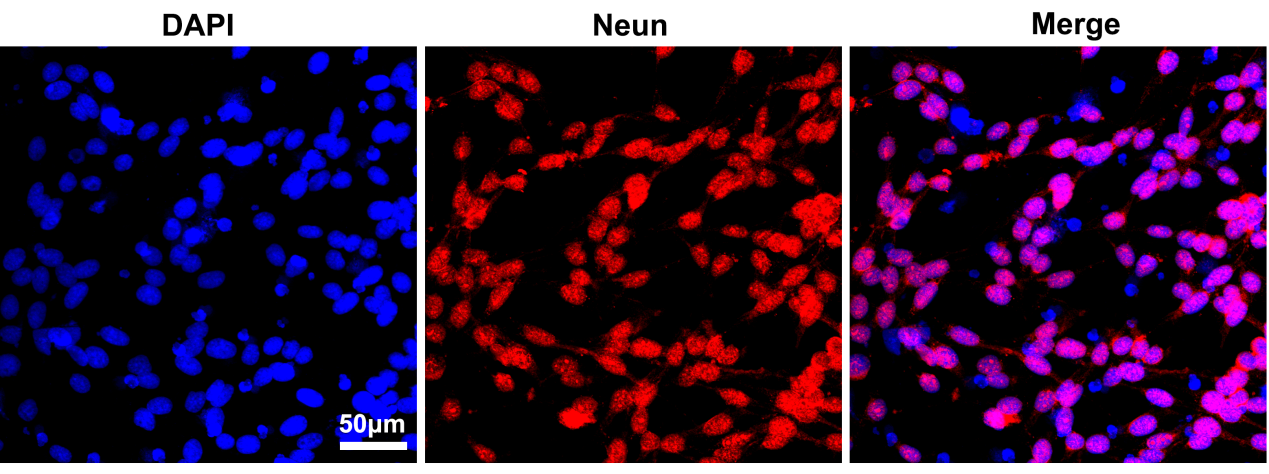
**

Figure 2. The purity of hippocampal neurons was identified by neuron-specific anti-NeuN antibody and DAPI double staining. Representative photomicrographs were captured under a confocal microscope (400×).


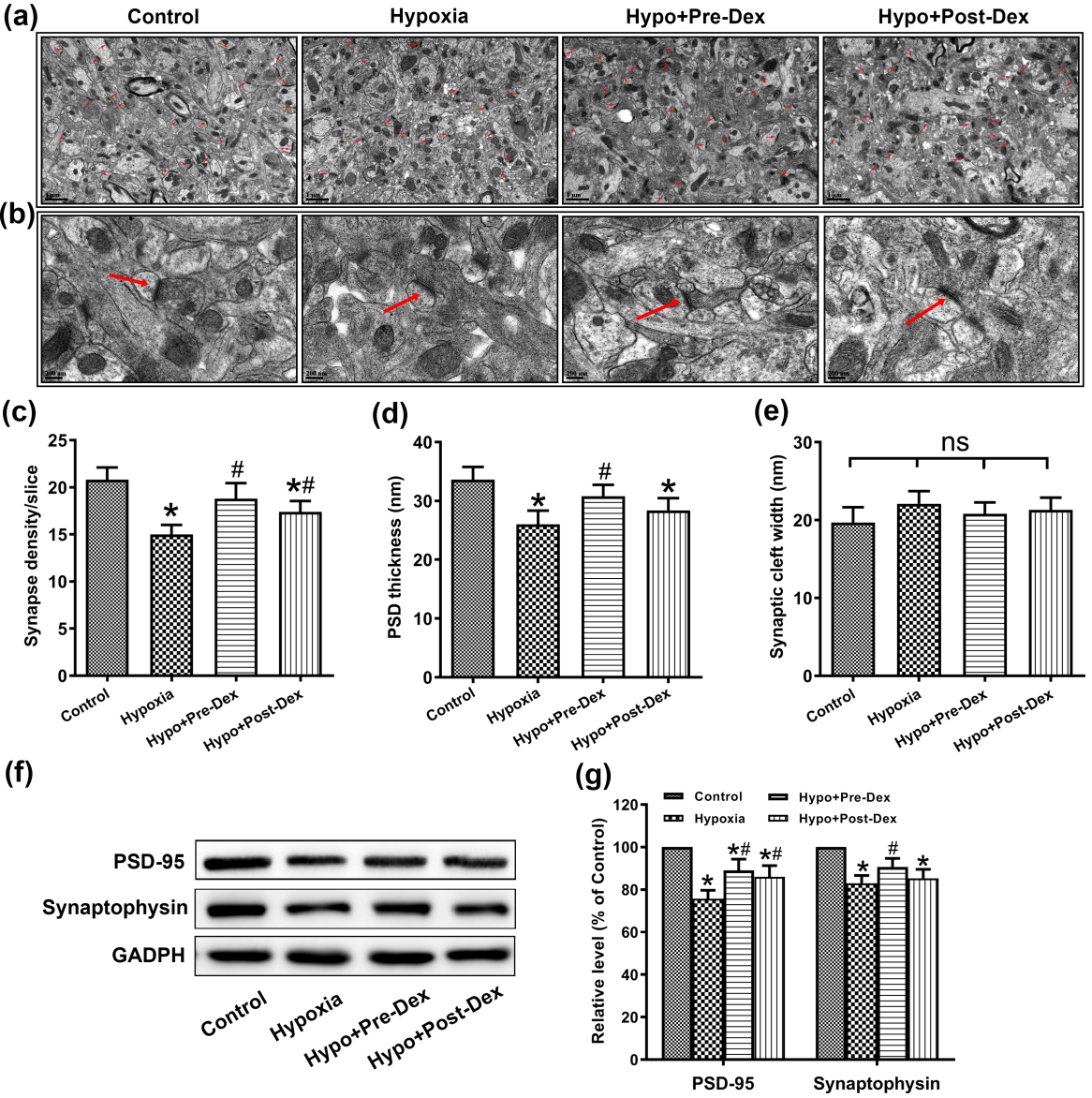


Figure 3: Dexmedetomidine partially attenuated hypoxia-induced synaptic loss in the cerebral cortex. Neonatal rats were treated with dexmedetomidine 30 min before or immediately after hypoxia exposure, and (a-e) synaptic ultrastructure changes in the prefrontal cortex were observed under TEM 28 days following hypoxia. (a) Representative photomicrograph (9700×) showing the differences in the number of synapses per slice among the four groups (the red arrows indicate the synapses). (b) Representative high-magnification photomicrograph (37000×) showing the differences in the thickness of PSD and the width of the synaptic cleft among the four groups (the red arrows indicate the synaptic linkage). (c-e) Quantification of synapse density, PSD thickness and synaptic cleft width from at least 20 sections among the four groups. The protein expression of PSD95 and synaptophysin was measured in the prefrontal cortex 28 days (f, g) after hypoxic insult by Western blot analysis. The levels of PSD-95 and synaptophysin expression are presented as the percentages of those in the Control group. The data are expressed as the mean ± SD, n=4. **P* < 0.05 vs. the Control group, ^#^*P* < 0.05 vs. the Hypoxia group. Hypo: hypoxia, Pre-Dex: dexmedetomidine pretreatment, Post-Dex: dexmedetomidine posttreatment.
